# Cerebral arterial air embolism in a child after intraosseous infusion

**DOI:** 10.1007/s10140-007-0681-2

**Published:** 2008-02-05

**Authors:** R. R. van Rijn, H. Knoester, A. Maes, A. C. van der Wal, B. Kubat

**Affiliations:** 1 Department of Radiology, Academic Medical Centre/Emma Children’s Hospital, Amsterdam, The Netherlands; 2 Department of Paediatric Intensive Care Unit, Academic Medical Centre/Emma Children’s Hospital, Amsterdam, The Netherlands; 3 Department of Pathology, Netherlands Forensic Institute, The Hague, The Netherlands; 4 Department of Pathology, Academic Medical Centre/Emma Children’s Hospital, Amsterdam, The Netherlands; 5 Working Group on Forensic Paediatric Medicine, Amsterdam, the Netherlands; 6 Department of Radiology, Academic Medical Centre Amsterdam, Meibergdreef 9, 1105 AZ Amsterdam Zuid-Oost, The Netherlands

**Keywords:** Tomography, Spiral computed, Embolism, Air, Intracranial embolism, Autopsy

## Abstract

Cerebral arterial air embolism (CAAE) has been reported as a rare complication of medical intervention. There has been one reported case of CAAE after the use of an intraosseous infusion (IO) system. We report on a case of CAAE after tibial IO infusion in a 7-month-old girl during resuscitation.

## Introduction

Cerebral arterial air embolism (CAAE) has been reported as a rare complication of medical intervention. We present a case of a 7-month-old girl who was admitted to our emergency department; post-mortem computed tomography (CT) showed a CAAE.

## Case report

A 7-month-old girl was admitted to our emergency department. She was known in our hospital because of prematurity (gestational age, 30 weeks) and an omphalocele, for which initial non-operative treatment with epithelialisation was instituted, and delayed surgical correction was planned. Outpatient follow-up showed no other medical problems. Psychomotor development was normal. Vaccinations were given according to the Dutch vaccination program.

According to the mother, the child awoke at 3 ‘o clock at night and, as what had happened before, her mother bottle fed her. During feeding, she started to vomit and became unresponsive. A 911 call was placed, and a friend of the family was called. The latter was first to arrive and transported the girl and her mother to the emergency department of the Academic Medical Centre Amsterdam. They arrived in our hospital approximately 45 min after the 911 call. Upon physical examination, a non-responsive child without spontaneous respiration or cardiac output was seen. Resuscitation was immediately started. During resuscitation, significant amounts of food were aspirated from her mouth and endotracheal tube. Several attempts were made to insert a central venous and arterial catheter, however, to no avail. To gain venous access, an intraosseous infusion (IOI) needle was placed in the right tibia.

**Fig. 1:**
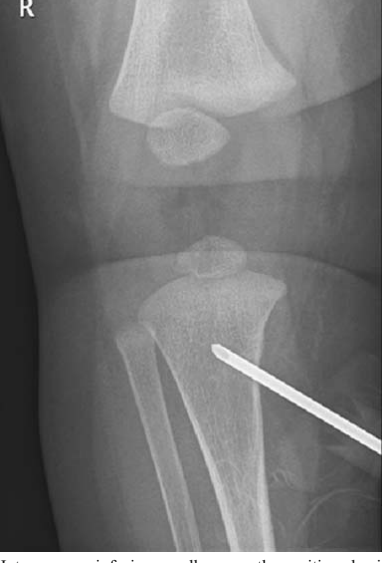
Intraosseous infusion needle correctly positioned within the proximal right tibial metaphysis

**Fig. 2:**
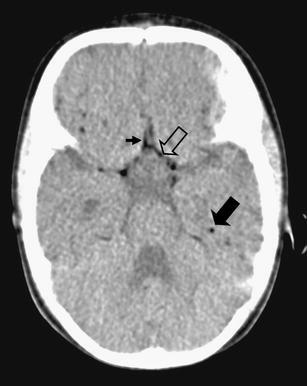
CT at the level of the circle of Willis showing air within the circle of Willis (*open arrow*) anterior cerebral artery (*small arrow*) and peripheral cerebral arteries (*solid arrow*)

At 5:00 a.m., she was pronounced deceased. Although the clinical history and findings during resuscitation suggested food aspiration, questions regarding the cause of death remained. Therefore, according to our battered child protocol, the standard radiographs, following the guidelines of the American College of Radiography were performed [1]. In addition, a head CT, which was a standard in our hospital in children under the age of 2 years with unexplained trauma/death, was performed within 1h after death. The skeletal radiographs showed no abnormalities, except for the IO infusion in the proximal right tibia (Fig. 1). Neither fractures nor signs of malnutrition were noted. The head CT, however, showed a considerable amount of air within the arterial circulation (Fig. 2).

**Fig. 3:**
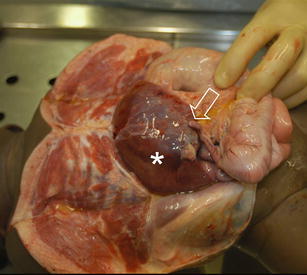
Omphalocele containing a large section of the right liver lobe (*asterisk*) and the ascending colon including the appendix (*arrow*)

**Fig. 4:**
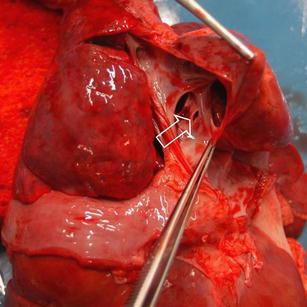
View of the atrial septum showing a persistent foramen ovale (*arrow*) and a fenestration (*arrowhead*)

**Fig. 5:**
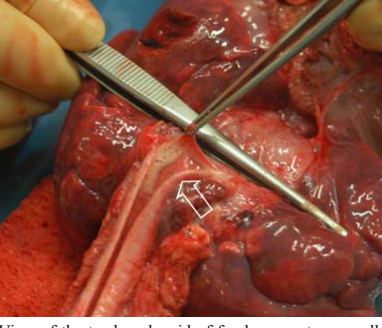
View of the trachea devoid of food remnants, a small amount of saliva is seen (*arrow*)

A full judicial autopsy was performed in The Netherlands Forensic Institute, as is mandatory in children with a possible non-natural cause of death. The body showed normal measurements (length, 64 cm, and 6,900 grams, both p50) and a known omphalocele. Autopsy showed that the omphalocele contained a large segment of the right liver lobe and the ascending colon including the appendix, with adhesion to the abdominal wall (Fig. 3). The intestines showed no signs of ischemia. Inspection of the heart revealed only minor congenital abnormalities, which consisted of a defect in the interatrial septum, fossa ovalis type with deficient flap valve, and slight hypertrabeculation of the ventricles, particularly the right ventricle (Fig. 4). The latter findings, however, were clearly insufficient for a diagnosis of ventricular non-compaction. There was some pulmonary oedema, possibly due to the vigorous fluid resuscitation. The trachea was without abnormalities and, in contrast to the clinical history, showed no signs of food aspiration (Fig. 5). Additional toxicological, microbiological and biochemical analyses of body fluids and tissues were all inconclusive.

As no distinct cause of death could be established, the cause of death remains undetermined.

## Discussion

CAAE is a rare finding; it has been described after medical instrumentation in trauma patients, after mechanical ventilation or resuscitation [2–8]. In case of vascular access procedures, CAAE can occur as a result from backflow, a right to left shunt, or shunting through the pulmonary capillary bed [9–11]. Backflow can be a result of manipulation of central lines (not necessarily IOI) and has been described in literature [12, 13]. Right to left shunting can be caused by an atrial septum defect (estimated incidence in adult patients 0.2–0.7 in 1, 000), a ventricular septum defect (incidence at birth ranging from 2–5%; however, 85–90% of these defects will close spontaneously by 1 year of age) and persistent foramen ovale (this has been reported to be present in up to 27% of autopsies) [14–16].

A special category are divers who suffer from decompression sickness after diving accidents [17, 18]. In severe cases, CAAE can develop, which is reported to be the second most common cause of death in divers [19]. Recently, an animal study with sheep was performed to test the hypothesis that artifacts caused by postmortem off gassing is at least partly responsible for the presence of gas within the vascular system and tissues of the cadaver after death associated with compressed air diving [20]. None of the control animals showed intravascular air on CT, respectively, 1 and 8 h after death; only after 24 h after the time of death, relatively small amounts of gas were seen. Although it is unknown how well sheep resemble 7-month-old infants, we assume that decay rates will not differ that much and certainly not to the extent that it can explain the amount of air visible on the CT scan.

In our case, the only vascular access had been IO infusion, which is a widely implemented technique and is part of the standard protocols, such as the Advanced Paediatric Life Support textbook. The advantage of IO infusion over peripheral vascular access is that it provides a rapid and reliable access to the systemic venous circulation in children [21–23]. It can be used in children of all ages, with the smallest child reported in literature weighing 800 g [24]. This has led to IO infusion having almost completely replaced saphenous cut down procedures in critically ill children in emergency situations. IO infusion has been used to introduce fluids, medication and even contrast media [25]. It is generally considered to be a safe technique with a reported complication rate of 1% [26]. Several complications resulting from the use of IO infusion have been reported; these consisted of extravasation in some cases leading to compartment syndrome (in rare cases leading to limb amputation), osteomyelitis, fracture, skin necrosis and fat embolism [24, 27–32].

Recently, investigators from the Berne Institute of Forensic Medicine (Switzerland) reported on a case of air embolism after resuscitation and IOI in a 4-month-old boy [33]. In their case, resuscitation was considered as the cause for CAAE; however, whole-body CT showed air in the lower extremity, which was used for IOI. As in our case, IO infusion was the only vascular access. Given the patent foramen ovale and atrial septal defect, leading to a right to left shunt, we feel that the only logical explanation for the CAAE is that air is introduced into the bloodstream via this route. To the best of our knowledge, our case is the second reported case of an air embolism after the use of an IO infusion.

Given the incidence of right-to-left shunts in the general population, this is a serious complication of IOI of which clinicians should be aware.
